# Burnout in Palliative Care Nurses, Prevalence and Risk Factors: A Systematic Review with Meta-Analysis

**DOI:** 10.3390/ijerph17207672

**Published:** 2020-10-21

**Authors:** Jose Luis Gómez-Urquiza, Luis Albendín-García, Almudena Velando-Soriano, Elena Ortega-Campos, Lucía Ramírez-Baena, María Jose Membrive-Jiménez, Nora Suleiman-Martos

**Affiliations:** 1 Faculty of Health Sciences, University of Granada, 18016 Granada, Spain; jlgurquiza@ugr.es; 2 La Chana Health Center, Granada Metropolitan District, 18071 Granada, Spain; 3 Andalusian Health Service, Virgen de las Nieves University Hospital, 18014 Granada, Spain; srtavelando@correo.ugr.es; 4 Psychology Department, University of Almería, 04120 Almería, Spain; elenaortega@ual.es; 5 Spanish Red Cross Nursing School, Sevilla University, 41009 Sevilla, Spain; luciarb@correo.ugr.es; 6 Ceuta University Hospital, National Institute of Health Management, Loma Colmenar s/n, 51003 Ceuta, Spain; mariajosemembrive@correo.ugr.es; 7 Faculty of Health Sciences, University of Granada, Cortadura del Valle s/n, 51003 Ceuta, Spain; norasm@ugr.es

**Keywords:** burnout, palliative care, nursing, occupational health, hospice, palliative care nursing, systematic review, meta-analysis

## Abstract

Palliative care nurses are exposed to hard situations, death, and duel feelings in their daily practice. These, and other work stressors, can favor burnout development. Thus, it is important to analyze the prevalence and risk factors of burnout in palliative care nurses and estimate its prevalence. A systematic review and meta-analysis was done with quantitative primary studies. *n* = 15 studies were included with *n* = 6 studies including information for the meta-analysis. The meta-analytic prevalence estimation of emotional exhaustion was 24% (95% CI 16–34%), for depersonalization was 30% (95% CI 18–44%) and for low personal accomplishment was 28% with a sample of *n* = 693 palliative care nurses. The main variables related with burnout are occupational variables followed by psychological variables. Some interventions to improve working conditions of palliative care nurses should be implemented to reduce burnout.

## 1. Introduction

Health professionals work with problems related to people’s health, which entails a high level of responsibility. Thus, work in the healthcare environment is considered one of the most prone to the development of stress and other related diseases. This continuous exposure to stress in the work environment, and its chronicity can end up producing the appearance of burnout syndrome [1].

Burnout is a three-dimensional syndrome that mainly occur in professionals who work for the public, such as healthcare professionals, and is characterized by emotional exhaustion (EE) or the feeling of exhaustion due to the physical and psychological load of the work; depersonalization (D) or tendency to treat people as objects and cynically; and low personal accomplishment (PA) or the feeling of dissatisfaction with the own work [2,3]. The main tool used worldwide for its evaluation is the Maslach Burnout Inventory (MBI) [2].

This syndrome has a high prevalence among nurses, being one of the most affected groups [4], and with different occupational, psychological, and sociodemographic factors that can influence its development [5,6,7]. For example, age has a negative correlation with EE and D, and men nurses tend to have higher D than women [5,8]. Some authors have reported negative consequences of burnout on nurses’ health such as insomnia, irritability, headaches, or related it with anxiety and depression [9,10]. In addition, there are also studies that report that burnout increases the number of mistakes at work, reduced the quality and safety of care, and that it favors sick leave or abandonment of the profession [11,12,13,14].

Another factor that can influence nurses burnout and that has been analyzed is the nurses’ work unit or the characteristic of that unit [15,16]. Daily practice, the type of diseases, patients and their prognosis, and the ethical issues will vary depending on the unit where the nurse work [17,18]. For this reason, it can be expected that the levels of burnout will be different depending on the work unit. For example, in gynecology units the most affected burnout dimension is low PA with a prevalence of 44% [19], while in pediatric oncology units it is EE with 37% [20] or in medical units D is the less affected with 24% [21].

In the case of palliative care nurses, it is a unit where people have a terminal prognosis, where bad news communication is more common and where the contact with family and patients is more continuous and close. For this reason, it is important to analyze palliative care nurses burnout. Previous review studies have focused their analysis on all palliative care professionals, mixing data from nurses and doctors [22,23,24] and providing limited quantitative data on mean values and prevalence of the syndrome and its related factors [22]. Others have been focused on analyzing compassion fatigue in all professionals of the unit [25] and none have done a meta-analysis to estimate the real impact of burnout in palliative care nurses. Thus, it is interesting to exclusively analyze the influence of burnout in palliative nurses and meta-analytically estimate the prevalence of the syndrome as has been done in other units.

The aim of the study was to analyze the levels and prevalence of EE, D, and PA in palliative care nurses, to know the related factors and to estimate a meta-analytical prevalence of burnout (EE, D, and low PA).

## 2. Materials and Methods

Following the Preferred Reporting Items for Systematic Reviews and Meta-Analyses (PRISMA) [26] a systematic review and meta-analysis was done.

### 2.1. Elegibility Criteria

Primary quantitative studies about the burnout syndrome in palliative care nurses published in English, Spanish, or Portuguese without restriction in the publication date were included. Studies with mixed samples without independent information for palliative care nurses were excluded.

### 2.2. Information Sources and Search

The next databases were used for the search: Pubmed (Medline), Scopus, CINAHL, and CUIDEN. The search equation with MeSH term was (burnout AND palliative care AND nurs*) OR (burnout AND Hospice and Palliative Care Nursing). The search was done in August 2020.

### 2.3. Study Selection and Critical Reading

The selection process was done in four steps. First, after eliminating duplicated studies with Mendeley^®^ (Elsevier Inc., New York, NY, USA), title and abstract were read. Then, the full-text of the remaining studies was read, followed by a critical reading. An inverse search was performed in the references of the selected studies. Two researchers did the selection process independently, consulting a third member in case of disagreement. For the critical reading, a checklist for descriptive quantitative studies was used [27] and for the level of evidence the Oxford Center for Evidence-Based Medicine recommendations were used [28].

### 2.4. Variables and Data Collection

A notebook was used for data collection including the following variables: authors of the study; country of the study; study design; sample; instrument for burnout evaluation; mean and prevalence of EE, D, and PA; and factors/variables related with EE, D, and PA. For the meta-analysis, the total sample and the sample with high EE, high D, and low PA were collected.

### 2.5. Data Analysis

A random effects meta-analysis about the prevalence of EE, D, and low PA were done with the StatsDirect^®^ software (StatsDirect Ltd., Cambridge, UK) [29]. The Egger test was used for the evaluation of publication bias and the I^2^ index for as heterogeneity test. Moreover, a sensitivity analysis was done before the analysis.

## 3. Results

### 3.1. Search and Characteristics of the Studies

**Figure 1:**
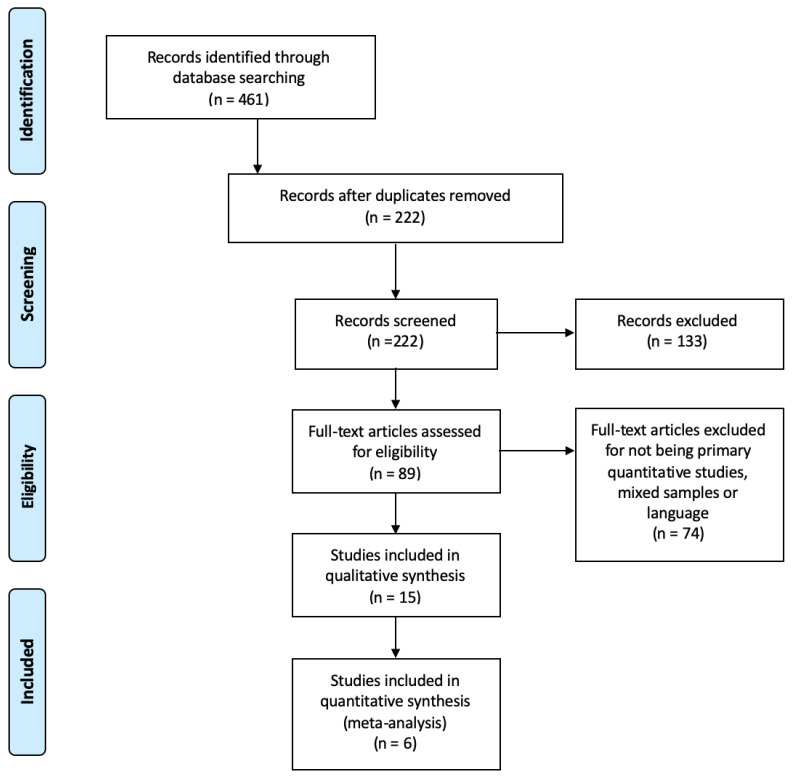
PRISMA flow diagram of studies selection.

The search showed a total of 461 studies. After eliminating the duplicates, the number was reduced to 222 studies. There were 89 studies that were selected after reading the title and abstract. Then, after reading the full-text, 15 studies [30,31,32,33,34,35,36,37,38,39,40,41,42,43,44] were selected applying the inclusion criteria and 6 of them had the necessary data for the meta-analysis. All studies passed the critical reading process. The selection process is shown in Figure 1.

All of the selected studies were quantitative and cross-sectional [30,31,32,33,34,35,36,37,38,39,40,41,42,43,44]. The 80% of the studies used the MBI-HSS for burnout measurement. All of the studies had a sample with more females (between 72% and 94.4%) and 73.3% of the studies were done in Europe. The level of evidence of all studies was 2c with a grade B of recommendation. The main characteristics and results of the studies are shown in Table 1.

### 3.2. Burnout Levels: EE, D, and Low PA Prevalences

EE prevalence showed a range between 7% [32] and 30.4% [36]. The mean score of EE from different studies showed a low level [34,40,43]. The prevalence rates are shown in Table 2.

In respect to high D prevalence, the highest was 65% in one study from Italy [32]. Low levels of D were shown from different studies with the mean score [34,40,43].

Regarding low PA it was present in 27% of the sample [32] while the mean score of PA from other studies showed a medium [34] and high [43] level.

Those studies that did not use the MBI, informed of a 26.8% of the sample with high burnout scores [33] and other showed a 16% with burnout [41]. Other authors showed that 65% of the palliative care nurses were in a good level of burnout, 29% in an alarming level, 1% in an acute crisis, and 5% had burnout [39].

The scores in EE and D were lower for palliative care nurses compared with the scores of nurses from internal medicine, oncology, or hematology, while the PA scores of palliative care nurses were the highest [34]. Other study informed of no difference in burnout levels between nurses and physicians [39], unlike than two studies with higher level of burnout [41] and D [43] in nurses. Another study showed that nurses developed burnout faster than physicians or physiotherapists [44].

### 3.3. Burnout Related Variables

Some studies analyzed the relation between sociodemographic variables and burnout. One study found that age had a negative correlation with burnout [37]. Senior nurses showed higher risk of burnout than junior nurses, but it was not significant on the multivariate analysis [38].

In respect to occupational variables, having workplace commitment, work freedom, possibilities for development at work, influence, and meaning of work were negatively correlated with burnout in palliative care nurses [31]. Commitment was also identified with a negative correlation in another study [33]. The same correlation between commitment and burnout was found in another study that also informed that workload had a positive correlation with burnout [31]. Workload was also identified by other authors as a burnout related factor, who said that working more than 8 h a day a risk factor [42]. On the other hand, other authors informed that satisfaction with family conciliation and the occupational situation and a higher salary were related with lower EE [36].

The relation with patients and their family is also correlated with burnout. One study informed that arguing with patients and their family was correlated with higher levels of EE and D, and with lower levels of PA [37]. The same relation was found with EE and arguing with colleagues and superiors [37].

Regarding psychological variables, being less extroverted and sociable was correlated with lower levels of EE, while lower levels of neuroticism were correlated with higher EE [35]. Other authors said that the feeling of having good health was negatively correlated with burnout in palliative care nurses [31]. Another study informed that EE was negatively correlated with having a meaning of life and positive affect [30], while psychological distress and negative affect were positively correlated with EE [30]. In this sense, having a meaning in life and a good self-esteem explained the variance of EE in a 3–16% [30]. Furthermore, burnout levels were lower in nurses with higher levels of psychological hardiness and psychological empowerment [33]. Another study informed that moderated levels of PA were shown by nurses that were not exceedingly open to changes [35].

The information about the burnout related factors is shown in Table 1.

### 3.4. Prevalence of High EE, High D, and Low PA Meta-Analysis Estimation.

**Figure 2:**
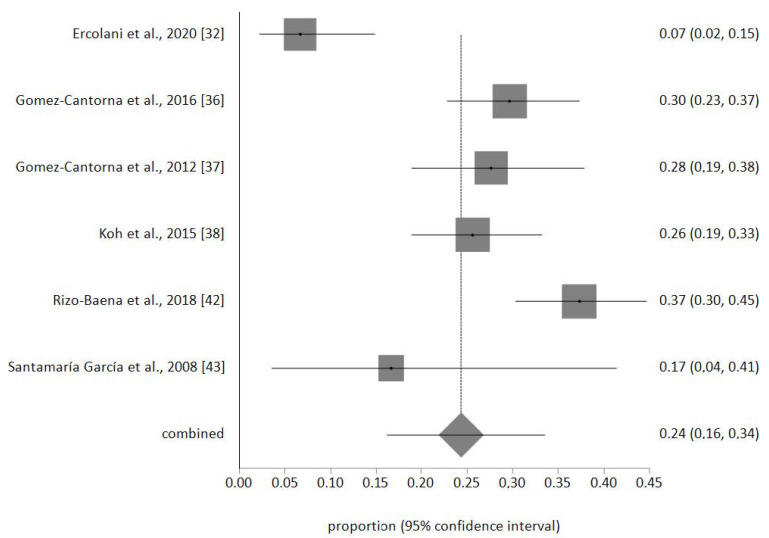
Forest plot of high EE.

**Figure 3:**
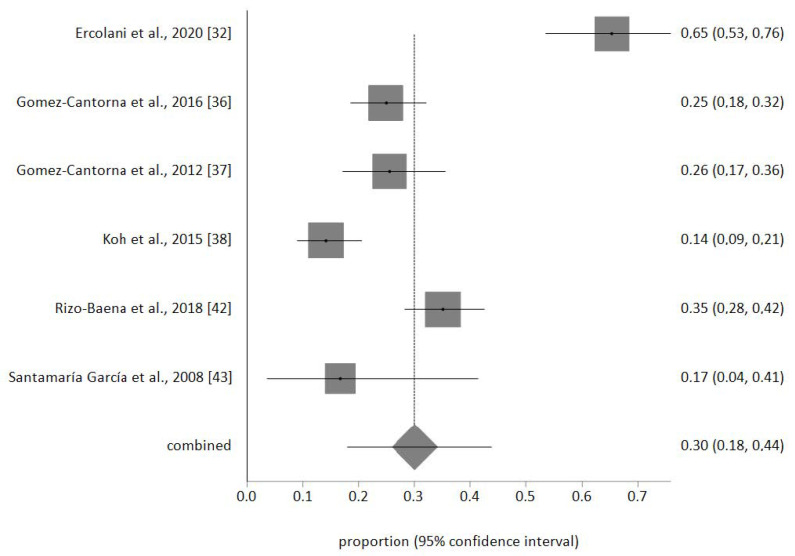
Forest plot of high D.

**Figure 4:**
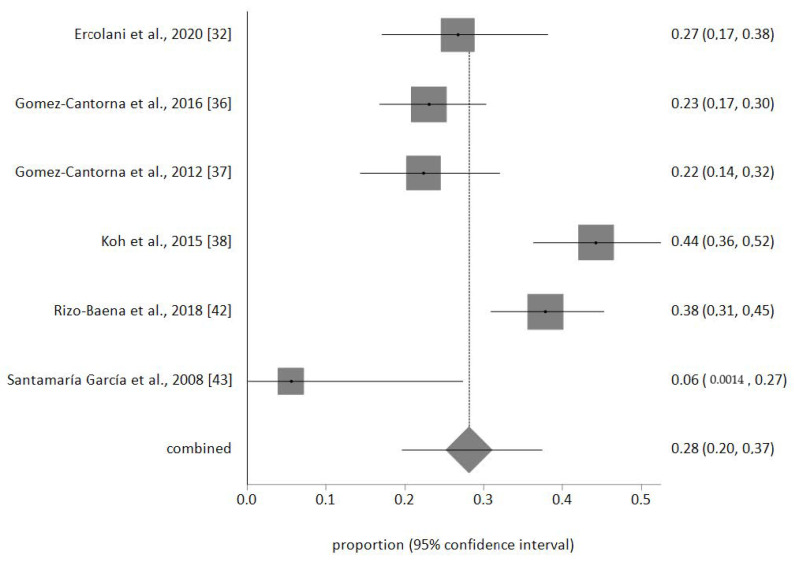
Forest plot of low PA.

The sample of palliative nurses included in the meta-analysis was *n* = 693. No publication biases were detected, and no study was removed after the sensitivity analysis. The prevalence meta-analytical estimation of high EE was 24% (95% CI 16–34%) as shown in Figure 2. For high D was 30% (95% CI 18–44%) (Figure 3) and for low PA was 28% (95% CI 20–37%) (Figure 4). The I^2^ was 85.1% for EE, 92.7% for D, and 84.3% for PA.

## 4. Discussion

The results of this review and meta-analysis have shown that between 24% and 30% of palliative care nurses are suffering one of the burnout components (EE, D, or low PA) and that the main variables related with burnout are occupational and psychological.

It would be important to analyze the burnout profile/state that palliative care nurses have experienced: engaged; ineffective (only feeling negatively about how well one is doing the job); overextended (singular concern with workload); disengaged (high in EE and D); and burnout (high EE and D, and low PA) [3], with being engaged, the best one; burnout, the worst one; and the other intermediate profiles being disengaged, the worst of those three.

Nurses with high levels of EE, D, and low PA may be due to spending more time exclusively with these kinds of patients, which can be very demanding and frustrating in clinical terms [23]. Since D is the most affected dimension, it is normal for it to affect the others, since working conditions are harsh and it ends up producing exhaustion and inefficiency with the patient [45].

These hard working conditions are the main risk factors for nurses dedicated to palliative care. Therefore, it is not surprising that services such as critical and emergency or internal medicine are among the most affected, since many of their patients are terminal [7,21,46]. In addition, these services have a higher work overload and scarcity of resources than other services, which worsens the conditions in which nurses carry out their work [47,48,49]. In fact, there are studies that show that up to 50% of nurses working in critical care units experience burnout. More specifically, it can be seen that they show higher levels in PA, and also high levels of EE and D due to the overload or scarcity of resources, among other factors [45].

Adverse working conditions are a challenge for nursing staff. The overload and scarcity of resources means that nurses are hired full time, with very long duties, rotating shifts, and fewer staff for each shift [50]. If we add low salaries, little autonomy, and a high nurse–patient ratio to the above, the staff develops job dissatisfaction [51]. This means that nurses have health problems such as irritability, stress, or insomnia and this leads to burnout, absenteeism, and abandonment of the profession [52,53]. In fact, there are studies that affirm that nurses have less job satisfaction the longer they work [54], which is why they also develop burnout [50].

The study has some limitations. The number of studies included in the meta-analysis is low because not all of the included studies had the necessary information to be included. Additionally, the studies analyzed come from different countries and the results may vary depending on the specific characteristics of nurses work environment in each country. Future research should analyze the effect of different strategies to improve the working conditions for reducing palliative care nurses burnout.

Regarding the clinical application of the results, it should be noticed that, for those nurses that have burnout, it is important to implement some intervention that have proven benefit for burnout like mindfulness [55]. Furthermore, for preventing burnout nursing managers from palliative care units should try to offer social support because it reduce stress and give emotional assistance [56]. They should also improve workload, family life conciliation, and interventions for promoting the psychological variables related with lower burnout scores.

## 5. Conclusions

The meta-analytical estimations of prevalence of emotional exhaustion, depersonalization, and low personal accomplishment in palliative care nurses are between 24% and 30%, indicating that an important part of them are being affected by the burnout syndrome. The most affected burnout dimension is depersonalization. The main related factors with burnout levels in palliative care nurses are occupational (workload, commitment, work environment, conciliation, and relations with patients and family) and psychological (extroversion, neuroticism, empowerment, meaning in life, and negative affect). Although not all palliative care nurses are affected by burnout, improvement in working environment and conditions and interventions for reducing burnout or preventing it are necessary.

## Figures and Tables

**Table 1:** Characteristics and main results of included studies (*n* = 15).

Authors, Year and Country	Sample	Instrument for Burnout Measurement	Mean (SD) of EE, D and PA	Burnout and Related Variables
Barnett et al., 2019 [30]. USA	*n* = 90 nurses. 94.4% females Mean age: 43.	MBI-HSS	-	EE correlations (r): -Presence of meaning of life (−0.26) * - Searching a meaning of life (0.11) -Psychological distress (0.49) * - Negative affect (0.34) * - Positive affect (−0.34) *
Diehl et al., 2020 [31]. Germany	*n* = 1316 nurses. 87.3% females. Age: 45.1% with 50 or more years.	Copenhagen Psychosocial Questionnaire	Burnout mean: 41.43 (17.61)	Burnout correlations (r): - Quantitative demands (0.442) * - Self-rated health (-0.554) * -Workplace commitment (-0.119) * - Degree of freedom (−0.248) * - Possibilities for development (−0.151) * - Influence at work (−0.215) * - Meaning of work (−0.257) *
Ercolani et al., 2020 [32]. Italy	*n* = 75 nurses. 72% females. Mean age: 37	MBI-HSS	EE: 12.7 D: 9.9 PA: 33.4	-
Frey et al., 2018 [33]. New Zealand.	*n* = 256 nurses. 92.2% females. Age: 51.2% between 35–54.	Professional Quality of Life Scale	Burnout: 23.26 (5.35)	Burnout correlations (r): - Secondary Traumatic Stress (0.613) * - Compassion satisfaction (−0.710) * - Commitment (−0.429) * - Control (−0.344) * - Challenge (−0.402) * - Psychological empowerment (−0.535) *
Gama et al., 2014 [34]. Portugal.	*n* = 59 nurses.	MBI-HSS	EE: 13.03 D: 3.42 PA: 38.63	-
Gomez-Cantorna et al., 2015 [35]. Spain.	*n* = 165 89% females Mean age: 37.	MBI-HSS	-	- Less extroverted and sociable nurses showed lower EE - Nurses with lower levels of neuroticism showed higher EE - Nurses that are not exceedingly open to changes showed moderate PA.
Gómez-Cantorna et al., 2016 [36]. Spain.	*n* = 165 nurses. 89% females Mean age: 37.	MBI-HSS	-	- EE was negatively correlated with working physical environment (−0.30) *. - The correlation with D and PA was not significant - Higher salary was related with lower EE. - Being satisfied with family life conciliation was related with lower EE. - Being satisfied with the occupational situation was related with lower EE.
Gomez-Cantorna et al., 2012 [37]. Spain.	*n* = 94 nurses. 87% females. Mean age: 39.53.	MBI-HSS	-	EE correlations (r): - To argue with patients (0.260) * - To argue with patients’ family (0.260) * - To argue with colleagues (0.271) * - To argue with superiors (0.348) * D correlations (r): - Age (−0.276) * - Job situation (−0.29) * - To argue with patients (0.243) * - To argue with patients´ family (0.424) * PA correlations (r): - Night shift (−0.208) * - To argue with patients (−0.306) * - To argue with patients´ family (−0.251) * - Number of nurses in the team (−0.253) *
Koh et al., 2015 [38]. Singapore	*n* = 156 nurses.	MBI-HSS	-	Senior nurses had higher risk of burnout than junior nurses
Pavelková & Buzgová, 2015 [39]. Czech Republic.	*n* = 139 nurses.	Burnout Measure	-	No difference in burnout levels between nurses and physicians
Pereira et al., 2012 [40]. Portugal.	*n* = 73 nurses. 88% females. Mean age: 33	MBI-HSS	EE: 19.63 D: 4.95 PA: 36.06	-
Pereira et al., 2014 [41]. Portugal.	*n* = 70 nurses.	MBI-HSS	-	Nurses had higher burnout than physicians.
Rizo-Baeza et al., 2018 [42]. Mexico.	*n* = 185 87% females.	MBI-HSS	-	Being a single parent, workload, working more than eight hours a day, self-care deficit and high professional quality of life was associated with burnout.
Santamaría-García et al., 2008 [43]. Spain.	*n* = 18 nurses.	MBI-HSS	EE: 17.45 (8.48) D: 4.24 (4.28) PA: 40.72 (6.69)	More D in nurses compared with physicians.
Santisteban Etxeburu et al., 2006 [44]. Spain.	*n* = 4 nurses.	MBI-HSS	EE: 19.5 D: 6.25 PA: 40.25	Nurses showed higher level of D than the other healthcare professionals and developed burnout sooner.

Note: D = Depersonalization; EE = Emotional Exhaustion; MBI-HSS = Maslach Burnout Inventory-Human Services; PA = Personal Accomplishment; * = *p* < 0.05.

**Table 2:** Prevalence of high EE, high D, and low PA in palliative care nurses.

Study	Sample	High EE	High D	Low PA
[32]	*n* = 75	7%	65%	27%
[36]	*n* = 165	30.4%	25.5%	23.6%
[37]	*n* = 94	28%	25%	22%
[38]	*n* = 156	26%	14.3%	44.8%
[42]	*n* = 185	37%	35.1%	37.8%
[43]	*n* = 18	16.7%	16.7%	5.6%

Note: D = Depersonalization; EE = Emotional Exhaustion; PA = Personal Accomplishment.
